# Morroniside Promotes PGC-1*α*-Mediated Cholesterol Efflux in Sodium Palmitate or High Glucose-Induced Mouse Renal Tubular Epithelial Cells

**DOI:** 10.1155/2021/9942152

**Published:** 2021-08-24

**Authors:** Junwei Gao, Peng Liu, Zhengri Shen, Ke Xu, Chenguang Wu, Feng Tian, Ming Chen, Lifan Wang, Ping Li

**Affiliations:** ^1^Institute of Basic Theory for Chinese Medicine, China Academy of Chinese Medical Sciences, Beijing 101300, China; ^2^Renal Division, Heilongjiang Academy of Chinese Medicine Sciences, Harbin 150036, China; ^3^Shunyi Hospital, Beijing Traditional Chinese Medicine Hospital, Beijing 101300, China; ^4^Beijing Key Lab Immune-Mediated Inflammatory Diseases, Institute of Clinical Medical Sciences, China-Japan Friendship Hospital, Beijing 100029, China

## Abstract

Lipid deposition is an etiology of renal damage caused by lipid metabolism disorder in diabetic nephropathy (DN). Thus, reducing lipid deposition is a feasible strategy for the treatment of DN. Morroniside (MOR), an iridoid glycoside isolated from the Chinese herb *Cornus officinalis* Sieb. et Zucc., is considered to be an effective drug in inhibiting oxidative stress, reducing inflammatory response, and countering apoptosis. To explore the protective mechanism of MOR in attenuating renal lipotoxicity in DN, we investigated the effect of MOR on an *in vitro* model of lipid metabolism disorder of DN established by stimulating mouse renal tubular epithelial cells (mRTECs) with sodium palmitate (PA) or high glucose (HG). Oil Red O and filipin cholesterol staining assays were used to determine intracellular lipid accumulation status. Results revealed that PA or HG stimulation inhibited the expressions of peroxisome proliferator-activated receptor *γ* coactivator 1*α* (PGC-1*α*), liver X receptors (LXR), ATP-binding cassette subfamily A member 1 (ABCA1), ABCG1, and apolipoprotein E (ApoE) in mRTECs as evidenced by western blot and quantitative real-time PCR, resulting in increased intracellular lipid deposition. Interestingly, MOR upregulated expressions of PGC-1*α*, LXR, ABCA1, ABCG1, and ApoE, thus reducing cholesterol accumulation in mRTECs, suggesting that MOR might promote cholesterol efflux from mRTECs via the PGC-1*α*/LXR pathway. Of note, silencing PGC-1*α* reversed the promotive effect of MOR on PA- or HG-induced cellular cholesterol accumulation. In conclusion, our results suggest that MOR has a protective effect on mRTECs under high lipid or high glucose conditions, which may be related to the promotion of intracellular cholesterol efflux mediated by PGC-1*α*.

## 1. Introduction

Diabetic nephropathy (DN) is one of the most common microvascular complications in type 2 diabetes patients. DN has become the most important cause of end-stage renal disease, accounting for about 25–40% of patients with diabetes [1, 2]. Renal lipotoxicity caused by lipid metabolism disorder is one of the pathogenic mechanisms of DN [3]. Specifically, excessive lipid deposition in the kidney aggravates the inflammatory reaction and insulin resistance, which in turn promotes production of reactive oxygen species, oxidative stress, and endoplasmic reticulum stress. Eventually, the result is impairment of the glomerular filtration barrier and renal failure [4].

Accumulation of intracellular cholesterol has been shown to be one of the mechanisms of renal injury caused by lipid metabolism disorder in DN [5]. Excessive cholesterol deposition in the kidney results from the imbalance of cholesterol homeostasis caused by decreased intracellular cholesterol efflux, increased intracellular cholesterol influx, and synthesis in the diabetes state [6, 7]. Damaged cholesterol efflux of renal tubular epithelial cells (RTECs) is one reason for cholesterol accumulation in the kidney [6]. Additionally, RTECs account for a large portion of the kidney mass and are the basis for maintaining electrolyte and water homeostasis [8]. Thus, the current study selected RTECs as the cell model. Lipotoxicity includes not only damage caused by excessive lipid deposition and oxidation but also the overload of de novo synthesis of lipids induced by a large amount of glucose in adipocytes and nonadipocytes [9]. Sodium palmitate (PA) has been shown to be associated with renal tubular cell damage, including apoptosis and inflammation [10]. It can enter the cells through free diffusion and active transport, resulting in lipid accumulation in RTECs [11]. Recent studies have also demonstrated that PA or high glucose (HG) stimulation induces lipid accumulation via lipid synthesis pathways in RTECs [12, 13].

Peroxisome proliferator-activated receptor *γ* coactivator 1*α* (PGC-1*α*) is a key regulator of mitochondrial biosynthesis and lipid metabolism [14]. PGC-1*α* activates a variety of nuclear receptors, including peroxisome proliferator-activated receptors (PPARs), liver X receptors (LXRs), and farnesoid X receptors (FXRs) [15, 16]. LXR, a member of the nuclear receptor superfamily, is highly involved in cholesterol efflux from cells by regulating the expression of ATP-binding cassette subfamily A member 1 (ABCA1) and ABCG1 [17, 18]. A previous study indicated that expression of the downstream ABCA1 gene is upregulated by activating LXR, which promotes cholesterol influx to high-density lipoprotein (HDL), and attenuates cholesterol deposition in the kidney [19]. Accordingly, we speculate that PGC-1*α* can affect renal lipid metabolism by regulating cholesterol efflux.

**Figure 1:**
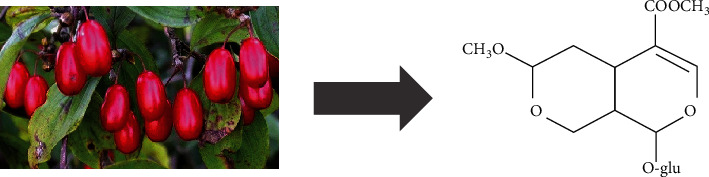
Morroniside is an active component of *Cornus officinalis* Sieb. et Zucc.

**Figure 2:**
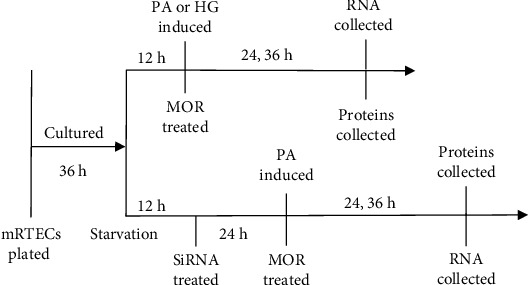
Experimental timeline for morroniside (MOR) treatments. After 12 hours of starvation under serum-free conditions, the mRTECs stimulated by PA or HG were treated with different concentrations of MOR. For siRNA knockdown experiments, the siRNA was added 24 hours before the addition of MOR. All assays were performed at two timepoints after MOR treatment.

Currently, treatment options for lipid metabolism disorder in DN are limited. The most widely used lipid-lowering drugs in the clinic include fibrates, statins, and niacin. However, the long-term and massive use of the above drugs may lead to gastrointestinal reactions, liver function damage, rhabdomyolysis, and other adverse reactions. Therefore, exploring new strategies for the treatment of lipid metabolism disorder is of great significance in the prevention and control of DN. *Cornus officinalis* is a traditional Chinese medicine that has a certain therapeutic effect on liver and kidney diseases [20]. Several studies in recent years have documented that morroniside (MOR), an iridoid glycoside derived from *Cornus officinalis* Sieb. et Zucc. (Figure 1), can promote the proliferation and differentiation of cells [21], regulate lipid synthesis [22], inhibit oxidative stress [23], reduce inflammatory response, and prevent apoptosis [24]. However, it is not clear by which mechanism MOR reduces renal cholesterol accumulation to prevent and delay the development of DN. In this study, an *in vitro* model of DN injury was established by stimulating mouse renal tubular epithelial cells (mRTECs) using PA or HG (Figure 2). We investigated the protective mechanism of MOR in DN and the mechanism of suppressing cholesterol efflux mediated by PGC-1*α*, so as to provide scientific evidence for the prevention and control of DN.

## 2. Materials and Methods

### 2.1. Reagents

MOR (molecular weight 406.38; purity > 98% by HPLC) was purchased from NatureStandard Biotechnology (Shanghai, China). The molecular formula of MOR is C_17_H_26_O_11_ (Figure 1).

### 2.2. Preparation of Sodium Palmitate (PA) and High Glucose (HG)

PA was procured from Sigma-Aldrich (St. Louis, MO, USA). PA was dissolved in deionized water at 70°C and diluted at 5 mmol/L with medium containing 1% bovine serum albumin (BSA). HG (35 mmol/L) culture medium was made from a specific proportion of 65 mmol/L D-(+)-glucose and basal culture medium. Both solutions of PA and HG were stored at 4°C before use.

### 2.3. Cell Culture

mRTECs were a gift from Professor Huiyao Lan at the Chinese University of Hong Kong. The cells were cultured at 37°C in a humidified 5% CO_2_ incubator, in Dulbecco's modified Eagle medium (DMEM) containing normal glucose (NG, 5.5 mmol/L) (Gibco; Grand Island, NY, USA), 10% fetal bovine serum (FBS) (Gibco), and 1% penicillin-streptomycin solution (Gibco) as previously reported [25].

### 2.4. Cell Viability Assay

The MTT assay was utilized to evaluate MOR on cell viability. The mRTECs in the logarithmic growth period were collected and seeded into a 96-well plate at 1 × 10^4^ cells/well. Subsequently, the cells were treated with different concentrations of MOR for 24 hours, and then, 20 *μ*L of MTT (5 mg/mL) (Solarbio; Beijing, China) was added and incubated for another 4 hours. The supernatant was discarded, and formazan products were dissolved in DMSO. The absorbance at 490 nm was measured using a microplate reader.

### 2.5. Cell Transfection of siRNA

The mRTECs were transfected with nontargeting negative control siRNA (control siRNA) or small interfering RNA (siRNA) targeting PGC-1*α* (PGC-1*α*-siRNA) using Lipofectamine 3000 (Mei5 Biotechnology; Beijing, China) according to the manufacturer's protocol. The sequences for the siRNA targeting PGC-1*α* gene (GenePharma; Shanghai, China) were sense 5′-GACGACAAAUCAGACAAGATT-3′ and antisense 5′-UCUUGUCUGAUUUGUCGUCTT-3′. The concentration of siRNA used was 20 *μ*mol/L. After 24 hours of siRNA transfection, the mRTECs were incubated with or without the indicated MOR concentrations [26]. PA was added to cells at the same time, except for the normal control group.

### 2.6. Oil Red O Staining and Filipin Staining

The mRTECs were seeded at 100,000 cells/well on coverslips in a 12-well plate. We treated cells stimulated by PA or HG for 36 hours with different concentrations of MOR. At the end of treatment, cells were fixed with 4% paraformaldehyde for 30 minutes and washed with 60% isopropanol. Next, cells on slides were stained with Oil Red O (Solarbio; Beijing, China) for 20 minutes at room temperature in the dark. After washing with phosphate-buffered saline (PBS) and counterstaining with hematoxylin, the images were acquired under a microscope (BX53 Olympus; Tokyo, Japan) at 400x magnification. For filipin staining, the cells were washed with PBS and fixed for 30 minutes with 4% paraformaldehyde. Then, cells were incubated with 1.5 mg/mL glycine for 10 min. Subsequently, the cells were stained with filipin working solution (125 *μ*g/mL) (Sigma-Aldrich). After several washes in PBS, the cells were mounted on a glass slide. The images were captured using a fluorescent microscope (BX53 Olympus) through an ultraviolet filter.

### 2.7. Measurement of Total Cholesterol (TC) and Total Triglyceride (TG) Levels

The mRTECs were collected and lysed with a buffer from the TC detection kit (Nanjing Jiancheng Bioengineering Institute, Nanjing, China). Next, the optical density (OD) values of the samples were obtained at 510 nm. Using the OD value, the cholesterol content can be calculated according to the formula in the manufacturer's instructions. Similarly, the amounts of TG in mRTECs were quantified and calculated using the corresponding detection kits (Nanjing Jiancheng Bioengineering Institute) according to the manufacturer's instructions.

### 2.8. Western Blotting Analysis

Western blot analysis was performed as described in our previous report with some modifications [11]. Briefly, the cell proteins were extracted using RIPA lysis buffer containing a phosphatase inhibitor cocktail (Mei5 Biotechnology). Membranes were incubated overnight with primary antibodies at 4°C, including PGC-1*α* (1 : 1000; sc-517380; Santa Cruz Biotechnology, Dallas, TX, USA), LXR (1 : 1000; sc-377260; Santa), ApoE (1 : 800; sc-13521; Santa), *β*-actin (1 : 10000; sc-47778; Santa), ABCA1 (1 : 500; ab18180; Abcam, Cambridge, MA, USA), and ABCG1 (1 : 1000; ab52617; Abcam). Then, the blots were probed with horseradish peroxidase-conjugated secondary antibodies (1 : 10000; Mei5 Biotechnology), and the bands were visualized using an ECL detection kit. The images were obtained using the gel imaging system program (iBright CL1000; Thermo Fisher Scientific, Waltham, MA, USA) and analyzed using ImageJ software (National Institutes of Health, Bethesda, MD, USA).

### 2.9. RNA Extraction and Quantitative Real-Time PCR

The total RNA from cells was isolated and reversed-transcribed into cDNA by RevertAid First Strand cDNA Synthesis Kit (Mei5 Biotechnology). Real-time PCR was carried out with a 7500 Fast Real-Time PCR System (Applied Biosystems, Waltham, MA, USA) using the UltraSYBR Green Mixture qPCR kit (Mei5 Biotechnology). Target gene expression was normalized by the expression of the internal control gene, *β*-actin. All primer sequences used in this study are shown in Table 1.

### 2.10. Statistical Analysis

All data were analyzed with the GraphPad Prism version 8.0 (GraphPad Prism Software, La Jolla, CA, USA), and quantitative data are presented as the mean ± standard error of mean (SEM). Data were analyzed using one-way analysis of variance (ANOVA) and Dunnett *t*-tests for multiple comparisons. *P* < 0.05 was considered statistically significant.

## 3. Results

### 3.1. Safety of MOR

**Figure 3:**
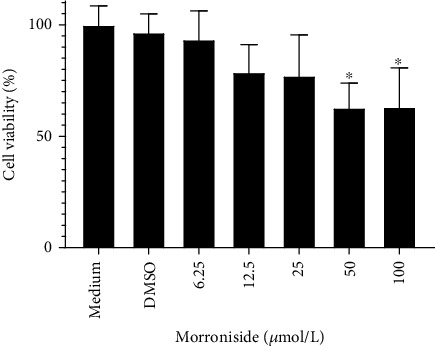
Effects of MOR on the proliferation of mouse renal tubular epithelial cells (mRTECs). Cells were cultured with medium containing different concentrations (6.25, 12.5, 25, 50, and 100 *μ*mol/L) for 24 hours, and the viability of cells was measured by the MTT method. ^∗^*P* < 0.05 vs. the medium group.

To investigate the impact of MOR on lipid accumulation, we tested the toxicity of MOR in mRTECs. Varying concentrations of MOR (6.25, 12.5, 25, 50, and 100 *μ*mol/L) were incubated with mRTECs for 24 hours, and the viability of mRTECs was measured using the MTT assay to evaluate the safety of MOR. Results indicated that cell viability was not affected at MOR concentrations of less than 25 *μ*mol/L. However, the cytotoxic effect on mRTECs was evident at MOR concentration above 50 *μ*mol/L (*P* < 0.05) (Figure 3).

### 3.2. MOR Alleviated Lipid Accumulation in PA- or HG-Stimulated mRTECs

**Figure 4:**
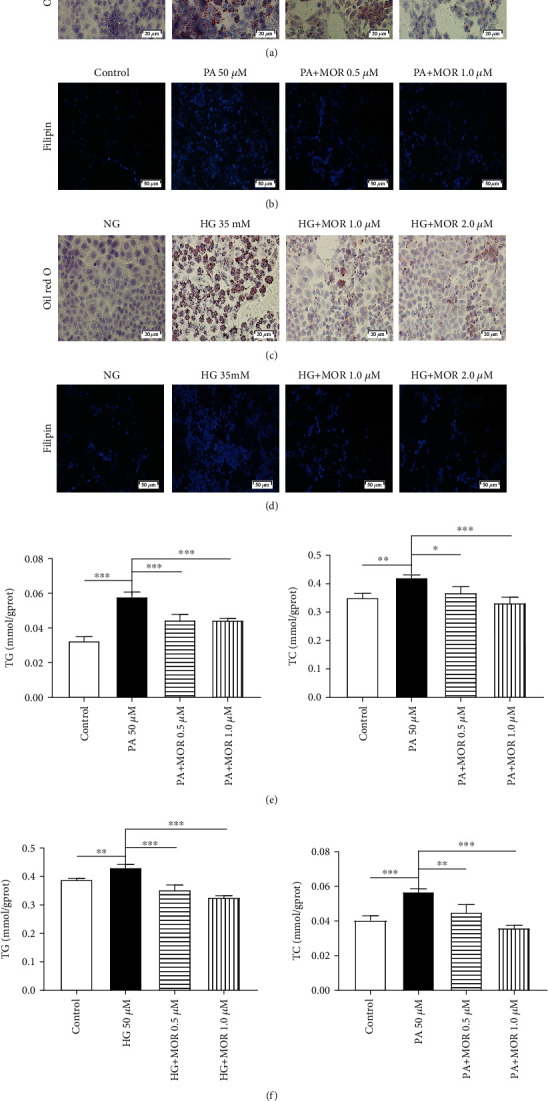
MOR attenuated cellular lipid and cholesterol accumulation in mRTECs stimulated by sodium palmitate (PA) or high glucose (HG). (a, c) Oil Red O staining reflected the lipid content of the mRTECs after 36 hours of PA or HG treatment (bar = 20 *μ*m). (b, d) Filipin cholesterol staining reflected cholesterol content of the mRTECs after 36 hours of PA or HG treatment (bar = 50 *μ*m). (e) Colorimetric analysis of total cholesterol (TC) and total triglyceride (TG) levels in mRTECs stimulated with PA for 36 hours. Data represent the mean ± SEM (*n* = 3). ^∗^*P* < 0.05, ^∗∗^*P* < 0.01, and ^∗∗∗^*P* < 0.001 compared with the PA group. (f) Colorimetric analysis of TC and TG levels in mRTECs stimulated with HG for 36 hours. Data represent the mean ± SEM (*n* = 3). ^∗^*P* < 0.05, ^∗∗^*P* < 0.01, and ^∗∗∗^*P* < 0.001 compared with the HG group.

Lipid and cholesterol accumulation in mRTECs was detected through Oil Red O staining and filipin staining, respectively (Figure 4). Lipid and cholesterol accumulation was induced by PA or HG after 36 hours and was significantly blocked by MOR treatment. Colorimetric analysis indicated that the TG and TC levels are significantly increased induced by PA (*P* < 0.001 and *P* < 0.01, respectively). Following treatment with MOR, the TG levels were markedly decreased compared with the PA group (*P* < 0.001). MOR treatment also decreased TC levels (*P*_MOR 0.5 *μ*M_ < 0.05 and *P*_MOR 1.0 *μ*M_ < 0.001, respectively) (Figure 4(e)). Likewise, the TG and TC levels are significantly increased induced by HG (*P* < 0.01 and *P* < 0.001, respectively). Following treatment with MOR, the TG levels were markedly decreased compared with the HG group (*P* < 0.001). MOR treatment also decreased TC levels (*P*_MOR 1.0 *μ*M_ < 0.01 and *P*_MOR 2.0 *μ*M_ < 0.001, respectively) (Figure 4(f)).

### 3.3. MOR Activating the PGC-1*α*/LXR Pathway in PA-Stimulated mRTECs

**Figure 5:**
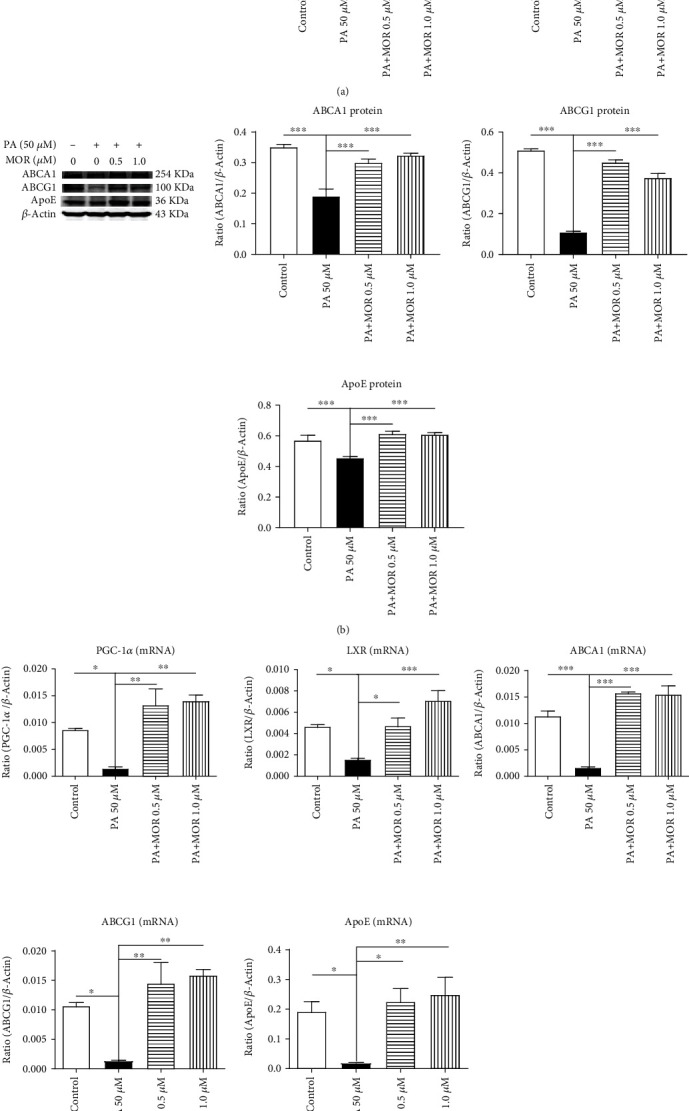
After treatment with MOR, expressions of PGC-1*α*, LXR, ABCA1, ABCG1, and ApoE were upregulated in mRTECs stimulated by PA. (a) Western blot analysis of PGC-1*α* and LXR expressions in mRTECs cultured for 24 hours. (b) Western blot analysis of ABCA1, ABCG1, and ApoE expressions in mRTECs cultured for 36 hours. (c) Real-time PCR analysis of PGC-1*α* and LXR expressions in mRTECs cultured for 24 hours. Real-time PCR analysis of ABCA1, ABCG1, and ApoE expressions in mRTECs cultured for 36 hours. Data represent the mean ± SEM (*n* = 3). ^∗^*P* < 0.05, ^∗∗^*P* < 0.01, and ^∗∗∗^*P* < 0.001 compared with the PA group.

It has been reported that cholesterol accumulation in the kidney results from damaged cholesterol efflux of RTECs [6]. Therefore, we investigated whether MOR promotes cholesterol efflux through the PGC-1*α*/LXR pathway to attenuate lipid deposition in PA-stimulated mRTECs. Western blot analysis identified that PGC-1*α* protein expression levels in mRTECs were upregulated after 24-hour exposure to 0.5 *μ*mol/L and 1.0 *μ*mol/L of MOR compared with the PA group (*P* < 0.01). LXR protein expression levels were consistent with PGC-1*α* protein expression levels (*P* < 0.05) (Figure 5(a)). Furthermore, ABCA1, ABCG1, and ApoE protein expression levels of mRTECs were significantly upregulated after 36-hour exposure to these two concentrations of MOR compared with the PA group (*P* < 0.001) (Figure 5(b)).

Compared with the PA group, PGC-1*α* mRNA expression levels of mRTECs were upregulated after 24-hour exposure to 0.5 *μ*mol/L and 1.0 *μ*mol/L of MOR (*P* < 0.01); LXR mRNA expression levels of mRTECs were upregulated after 24-hour exposure to both concentrations of MOR (*P*_MOR 0.5 *μ*M_ < 0.05 and *P*_MOR 1.0 *μ*M_ < 0.001, respectively); ABCA1 mRNA expression levels of mRTECs were upregulated after 36-hour exposure to both concentrations of MOR (*P* < 0.001); ABCG1 mRNA expression levels of mRTECs were upregulated after 36-hour exposure to both concentrations of MOR (*P* < 0.01); ApoE mRNA expression levels of mRTECs were upregulated after 36-hour exposure to both concentrations of MOR (*P*_MOR 0.5 *μ*M_ < 0.05 and *P*_MOR 1.0 *μ*M_ < 0.01, respectively) (Figure 5(c)).

Hence, MOR treatment significantly increased the PGC-1*α* expression at both the protein and mRNA levels in mRTECs. In addition, stimulation of cells with PA resulted in a significant downregulation of the mRNA and protein expressions of PGC-1*α* downstream target gene, and its expressions were restored upon treatment with MOR. These data indicate that MOR attenuates lipid deposition in the kidney, at least partly, through activation of PGC-1*α* and increasing its target gene transcription.

### 3.4. MOR Activating the PGC-1*α*/LXR Pathway in HG-Stimulated mRTECs

**Figure 6:**
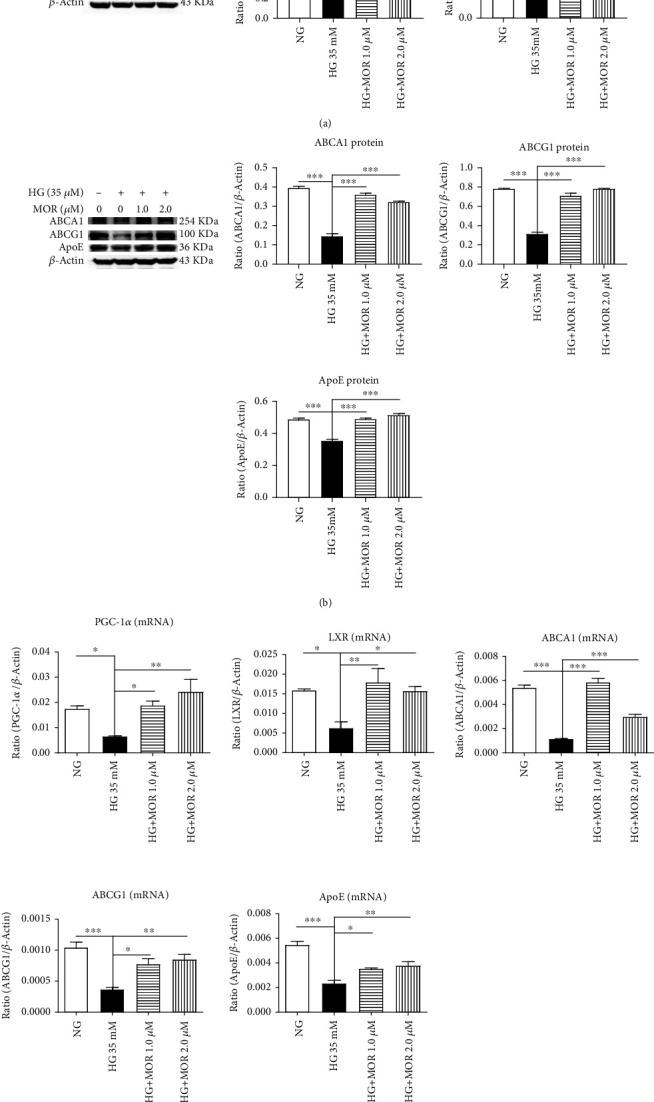
After treatment with MOR, expressions of PGC-1*α*, LXR, ABCA1, ABCG1, and ApoE were upregulated in mRTECs stimulated by HG. (a) Western blot analysis of PGC-1*α* and LXR expressions in mRTECs cultured for 24 hours. (b) Western blot analysis of ABCA1, ABCG1, and ApoE expressions in mRTECs cultured for 36 hours. (c) Real-time PCR analysis of PGC-1*α* and LXR expressions in mRTECs cultured for 24 hours. Real-time PCR analysis of ABCA1, ABCG1, and ApoE expressions in mRTECs cultured for 36 hours. Data represent the mean ± SEM (*n* = 3). ^∗^*P* < 0.05, ^∗∗^*P* < 0.01, and ^∗∗∗^*P* < 0.001 compared with the HG group.

Similarly, we found a significant upregulation of PGC-1*α* protein expression levels following 24-hour exposure to 1.0 *μ*mol/L and 2.0 *μ*mol/L of MOR compared with the HG group (*P* < 0.001 and *P* < 0.01, respectively); LXR protein expression levels were consistent with PGC-1*α* protein expression levels (*P* < 0.001) (Figure 6(a)). Moreover, ABCA1, ABCG1, and ApoE protein expression levels of mRTECs were significantly upregulated after 36-hour exposure to these two concentrations of MOR compared with the HG group (*P* < 0.001) (Figure 6(b)).

Compared with the HG group, PGC-1*α* mRNA expression levels of mRTECs were upregulated after 24-hour exposure to 1.0 *μ*mol/L and 2.0 *μ*mol/L of MOR (*P* < 0.05 and *P* < 0.01, respectively); LXR mRNA expression levels of mRTECs were upregulated after 24-hour exposure to these two concentrations of MOR (*P*_MOR 1.0 *μ*M_ < 0.01 and *P*_MOR 2.0 *μ*M_ < 0.05, respectively); ABCA1 mRNA expression levels of mRTECs were upregulated after 36-hour exposure to both concentrations of MOR (*P* < 0.001); ABCG1 mRNA expression levels of mRTECs were upregulated after 36-hour exposure to both concentrations of MOR (*P*_MOR 1.0 *μ*M_ < 0.05 and *P*_MOR 2.0 *μ*M_ < 0.01, respectively); ApoE mRNA expression levels of mRTECs were upregulated after 36-hour exposure to both concentrations of MOR (*P*_MOR 1.0 *μ*M_ < 0.05 and *P*_MOR 2.0 *μ*M_ < 0.01, respectively) (Figure 6(c)).

### 3.5. Silencing PGC-1*α* Reversed the Protective Effect of MOR on PA-Induced Lipid Accumulation in mRTECs

**Figure 7:**
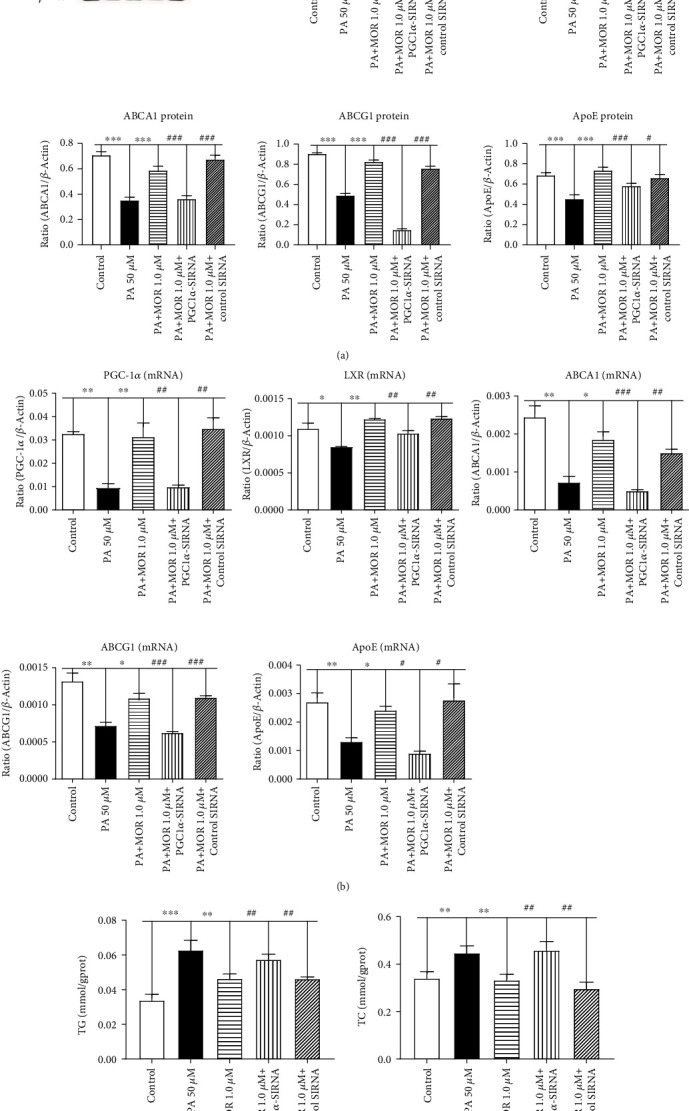
Silencing PGC-1*α* significantly suppressed the inhibitory effect of MOR on PA-induced total cholesterol levels in mRTECs. (a) Western blot analysis of PGC-1*α*, LXR, ABCA1, ABCG1, and ApoE expressions in mRTECs cultured for 36 hours and then exposed to PA (sodium palmitate 50 *μ*M), PA+M (PA 50 *μ*M + MOR 1.0 *μ*M), PA+M+P (PA 50 *μ*M + MOR 1.0 *μ*M + PGC1*α*‐siRNA), or PA+M+C (PA 50 *μ*M + MOR 1.0 *μ*M + control siRNA). (b) Real-time PCR analysis of PGC-1*α* and LXR expressions in mRTECs cultured for 24 hours. Real-time PCR analysis of ABCA1, ABCG1, and ApoE expressions in mRTECs cultured for 36 hours. (c) Colorimetric analysis of TC and TG levels in mRTECs cultured with PGC1*α*-siRNA for 36 hours. Data represent the mean ± SEM (*n* = 3). ^∗^*P* < 0.05, ^∗∗^*P* < 0.01, and ^∗∗∗^*P* < 0.001 compared with the PA group; ^#^*P* < 0.05, ^##^*P* < 0.01, and ^###^*P* < 0.001 compared with the PA+M+P group.

To determine whether promotion of renal cholesterol efflux by MOR was associated with activation of PGC-1*α*, we used siRNA to knock down the expression of PGC-1*α*. Silencing PGC-1*α* significantly abolished activation of MOR on PA-induced mRNA expression levels of LXR, ABCA1, ABCG1, and ApoE in mRTECs (*P* < 0.01, *P* < 0.01, *P* < 0.001, *P* < 0.001, and *P* < 0.05, respectively) (Figure 7(a)). The protein expression levels were consistent with the respective mRNA expression levels (*P* < 0.001, *P* < 0.05, *P* < 0.001, *P* < 0.001, and *P* < 0.001, respectively) (Figure 7(b)). Moreover, silencing PGC-1*α* significantly increased TG and TC levels compared with the PA+MOR group (*P* < 0.01) (Figure 7(c)). Thus, MOR may exert protective effects through PGC-1*α*-mediated dependent mechanisms, so as to improve renal lipid deposition. MOR alleviated renal lipid deposition by possibly exerting protective effects through PGC-1*α*-mediated dependent mechanisms.

## 4. Discussion

Findings from the present study provide a new understanding that cholesterol accumulation in mRTECs appears to be linked to inhibition of PGC-1*α*-mediated cholesterol efflux. Furthermore, treatment with MOR promoted renal cholesterol efflux, which was correlated with reducing PA- or HG-induced mRTEC lipid deposition via the PGC-1*α*/LXR pathway.

It is well known that the pathogenesis of DN is closely associated with multiple complex molecular mechanisms triggered by long-term hyperglycemia, and its occurrence and development are the results of multiple factors such as genetic factors, hemodynamic changes, abnormal glucose and lipid metabolism, and excessive activation of related cytokines [3]. Ruan et al. put forward the hypothesis of lipid nephrotoxicity and established the concept that dyslipidemia may lead to kidney disease. The hypothesis suggests that constant urinary albumin loss stimulates compensatory synthesis of large amounts of lipoproteins in the liver leading to hyperlipidemia and lipid-mediated kidney damage, thereby aggravating the progression of glomerular and tubulointerstitial diseases [27]. Hyperlipidemia accompanied by hyperglycemia can contribute to the accumulation of lipids in glomeruli and proximal renal tubules and accelerate the progression of kidney disease in animal studies [28]. Accordingly, further in-depth exploration into the pathogenesis of lipid metabolism disorders in DN has important implications for delaying the progression of DN.

The PGC-1*α* gene, located on chromosome 4p15.1, consists of 13 exons and encodes a 91 kDa protein containing 798 amino acids [29]. It is highly involved in regulating multiple energy metabolic processes, including gluconeogenesis, lipogenesis, and fatty acid oxidation [30]. As a key regulator of mitochondrial biosynthesis and lipid metabolism, PGC-1*α* is mainly expressed in tissues and organs with high energy demand and rich in mitochondria, such as the heart, skeletal muscle, kidney, liver, and brown adipose tissue. After the heart, the kidney is the organ with the highest PGC-1*α* expression [14, 31]. Through analysis of previous studies, we found that regulation of PGC-1*α* on glucose and lipid metabolism is highly correlated with the occurrence of DN. For example, Sharma et al. showed that the expression level of PGC-1*α* in renal biopsy tissues of patients with DN was lower than in patients with minimal change nephropathy, suggesting that the low expression of PGC-1*α* may be an important step in the pathogenesis of DN [32]. Tran et al. reported that PGC-1*α* knockout mice had more severe tubular injury and impaired renal function, while mice with overexpression of PGC-1*α* in renal tubules had less severe acute kidney injury, better renal perfusion, and faster recovery of renal function [33]. Another research has further indicated that resveratrol reverses renal lipid deposition and suppresses renal cell apoptosis and oxidative stress by activating the AMPK/SIRT1/PGC1-*α* pathway, thus achieving a protective effect against DN [34]. *In vitro* experiments revealed that PGC1-*α* inhibits the formation of foam cells and prevents oxidized low-density lipoprotein (oxLDL) from entering the vascular wall, thereby exerting an antiatherogenic effect [35]. PGC-1*α* has previously been shown to be a coactivator of LXR. LXRs (LXR*α* and LXR*β*) belong to the nuclear receptor superfamily of ligand-activated transcription factors, regulating genes related to the cellular cholesterol efflux pathway, including ABCA1, ABCG1, and ApoE [36]. Intracellular cholesterol homeostasis is regulated by cholesterol synthesis, endocytosis mediated by LDL receptor, and cholesterol efflux mediated by HDL apolipoprotein in most cells [37]. Furthermore, ABCA1 and ABCG1 mediate cholesterol efflux from cells to apolipoprotein A-I (ApoA-I) and HDL [18, 38]. ApoE, a 34 kDa glycoprotein, is implicated in the formation of HDL, acting both as a ligand for receptor-mediated lipoprotein clearance and a receptor for cholesterol efflux from peripheral cells [39]. Moreover, the antiatherogenic effects of ApoE are associated with the fact that ApoE can promote the efflux of cholesterol from peripheral cells to HDL to some extent and that it is also involved in reverse cholesterol transport [40].

Other *in vitro* experiments suggest that PGC-1*α* expression is downregulated and suppressed in mRTECs stimulated by PA or HG, and treatment with MOR markedly upregulated and activated expression of PGC-1*α* and the downstream target genes LXR, ABCA1, ABCG1, and ApoE, thereby facilitating cholesterol efflux from mRTECs. In addition, silencing PGC-1*α* significantly inhibited activation of MOR on PA-induced expressions of LXR, ABCA1, ABCG1, and ApoE in mRTECs. Previous studies have elucidated that MOR downregulates expressions of RAGE, p38MAPK, and NF-*κ*B through the AGEs/RAGE signaling pathway, so as to alleviate oxidative stress-induced damage and exert a protective effect against diabetic renal injury [41]. Moreover, MOR reduces triglyceride and cholesterol levels by downregulating the expressions of SREBP-1 and SREBP-2 in the kidneys of db/db mice, thus improving abnormal lipid metabolism in the kidneys [22]. The above results are in agreement with our *in vitro* cell model findings.

**Figure 8:**
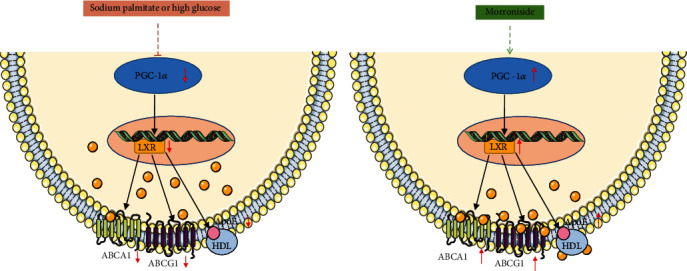
Schematic model of the PGC-1*α*/LXR pathways in mRTECs and the protective effect of MOR on PA or HG-induced lipid accumulation in mRTECs. MOR can activate the activities of ABCA1, ABCG1, and ApoE with PGC-1*α* as the central target to inhibit lipid accumulation in mRTECs.

In conclusion, this present study revealed that morroniside (MOR), an active ingredient in the Chinese herb *Cornus officinalis* Sieb. et Zucc., can activate the activities of ABCA1, ABCG1, and ApoE with PGC-1*α* as the central target to inhibit lipid deposition in cells and improve lipid metabolism disorder in the kidney, thus preventing or delaying the occurrence and development of DN (Figure 8). These data inspire us to rerecognize the mechanism by which MOR is involved in inhibiting cholesterol efflux and the potential application of MOR in the treatment of DN.

## Figures and Tables

**Table 1:** Primers used for quantitative real-time PCR.

Gene	Species	Forward primer (5′-3′)	Reverse primer (5′-3′)
PGC-1*α*	Mouse	CCGAGAATTCATGGAGCAAT	TTTCTGTGGGTTTGGTGTGA
LXR	Mouse	CTGCAGGACAAAAAGCTTCC	CCCTTCTCAGTCTGCTCCAC
ABCA1	Mouse	CCAGACAGTTGTGGATGTGG	GACCTCGCTCTTCCTTCCTT
ABCG1	Mouse	GGGTCTGAACTGCCCTACCT	TACTCCCCTGATGCCACTTC
ApoE	Mouse	GGTTCGAGCCAATAGTGGAA	ATGGATGTTGTTGCAGGACA

## Data Availability

All datasets generated for this study are included in the article.
